# Chemical Compositions of *Ligusticum chuanxiong* Oil and Lemongrass Oil and Their Joint Action against *Aphis citricola* Van Der Goot (Hemiptera: Aphididae)

**DOI:** 10.3390/molecules21101359

**Published:** 2016-10-12

**Authors:** Chao Zhang, Runqiang Liu, Jun He, Zhiqing Ma, Xing Zhang

**Affiliations:** 1 Research and Development Center of Biorational Pesticide, Northwest Agriculture & Forestry University, Yangling 712100, Shaanxi, China; zhangchao1990@nwsuaf.edu.cn (C.Z.); lrqiang1981@163.com (R.L.); hejun1963@yeah.net (J.H.); zhxing1952@gmail.com (X.Z.); 2 Shaanxi Research Center of Biopesticide Engineering & Technology, Yangling 712100, Shaanxi, China

**Keywords:** lemongrass oil, *Ligusticum chuanxiong* oil, *Aphis citricola* van der Goot, botanical aphicides

## Abstract

In order to develop novel botanical insecticides, the joint action of *Ligusticum chuanxiong* oil (LCO) and lemongrass oil (LO) against *Aphis citricola* van der Goot was determined systematically indoors and outdoors. The chemical profiles of LCO and LO as determined by gas chromatography–mass spectrometry (GC-MS) analysis revealed that the main compounds from LCO were (*Z*)-ligustilide (44.58%) and senkyunolide A (26.92%), and that of LO were geranial (42.16%) and neral (32.58%), respectively. The mixture of LCO and LO showed significant synergy against *A. citricola*, with a common-toxicity coefficient (CTC) value of 221.46 at the optimal ratio of LCO to LO (4:1, *w*:*w*). Based on the results of solvents and emulsifiers screening, *L. chuanxiong* oil·Lemongrass oil 20% emulsifiable concentrate (20% LCO·LO EC) was developed, and its stability was confirmed with tests of cold and thermal storage. Field trials indicated that the insecticidal activity of the diluted 20% LCO·LO EC (1000 fold dilution) was comparable to conventional pesticide (20% imidacloprid EC) on *A. citricola* seven days after application. Thus, the mixture of LCO and LO has the potential to be further developed as a botanical pesticide.

## 1. Introduction

Aphids (Hemiptera: Aphididae) are among the most destructive pests, widely classified in more than 4300 described species [1]. Aphids threat crops by aphids through feeding on plants, transmitting plant pathogenic viruses, and secreting honeydew. This could lead to secondary fungal infection and inhibits photosynthesis [2,3,4]. Aphids have become one group of the most serious pests in agriculture production because of rapid reproduction and specific feeding habits. Approximately, hundreds of millions of dollars in crop losses are caused by aphids annually [5].

In the past few decades, various insecticides have been used to control aphids [6]. As a result of frequent chemical applications, aphids have developed a high resistance to numerous commonly used insecticides in many agricultural areas. For example, the green peach aphid, *Myzus persicae* (Hemiptera: Aphididae), has developed resistance to at least 70 different synthetic compounds [7], and cotton aphid, *Aphis gossypii* Glover (Hemiptera: Aphididae), is resistant to carbamate, organophosphorus (OP), pyrethroid, and neonicotinoid insecticides [8,9].

Certain plant essential oils are widely used in the flavoring and fragrance industries and in aromatherapy. They can be obtained by expression, fermentation, enfleurage, or extraction, while steam distillation is most commonly used in commercial production. Moreover, some aromatic plants have traditionally been used to protect stored products, and the development potential of plant essential oils for broad-spectrum pest management has been realized for several decades [10]. Both antimicrobial efficacy and insecticidal effects of the essential oils have been reported [11,12,13]. Recent investigations in several countries have confirmed that some plant essential oils not only repel insects, but also have contact and fumigant activities against specific pests and fungicidal actions against some important plant pathogens [14]. Essential oils of cumin (*Cuminum cyminum* L.), anise (*Pimpinella ansium* L.), oregano (*Origanum syriacum* L.), and eucalyptus (*Eucalyptus dives*) were confirmed to be effective as fumigants against two greenhouse pests, the cotton aphid (*Aphis gossypii*) and the carmine spider mite (*Tetranychus cinnabarinus*) [15].

*Ligusticum chuanxiong* Hort. (Apiaceae), Chinese lovage, has been employed as a traditional Chinese medicine in folk remedies for long [16]. Twenty compounds have been identified in the essential oil from *L. chuanxiong*, and the major compounds are phenolics [17]. As a consequence, it is widely applied in food preparation as an antioxidant [16]. Moreover, *L. chuanxiong* oils (LCO) have been found to possess insecticidal activity against maize weevils, *Sitophilus zeamais* (Coleoptera: Curculionidae) [18].

*Cymbopogon citratus* (Poaceae), lemongrass, is one of the most commonly used plants for the treatment of nervous and gastrointestinal disturbances, and the antibacterial properties of its essential oil have been studied [19]. Lemongrass oil (LO), an important oil, has been shown to reduce aflatoxin formation and impede fungal growth of *Aspergillus flavus* Link. in stored rice [19,20]. The quality of LO is generally determined by its citral content, and citral (3,7-dimethyl-2,6-octadienal) consists of cis-isomer geranial and the trans-isomer neral [21,22].

However, little research in field trials and formulation preparations of plant essential oils for aphid management has been reported. The objective of this study was to assess in more detail the potential of LCO and LO to control *Aphis citricola* (Hemiptera: Aphididae). This study is mainly focused on the bioactivities and the synergistic effect of LCO and LO against *A. citricola* indoors and outdoors. A LCO·LO 20% emulsifiable concentrate (LCO·LO 20% EC) was successfully used in field trials with a significant effect against *A. citricola*.

## 2. Results

### 2.1. Toxicity of LCO and LO against Aphis citricola

Bioassays of 20% LCO EC and 20% LO EC against *A. citricola* were conducted, and the results are shown in Table 1. Both LCO and LO showed high toxicity against *A. citricola*, with LC_50_ values of 128.8 and 169.6 mg/L, respectively, 24 h after treatment.

### 2.2. Identification and Quantification of Compounds from LCO and LO

**Figure 1:**
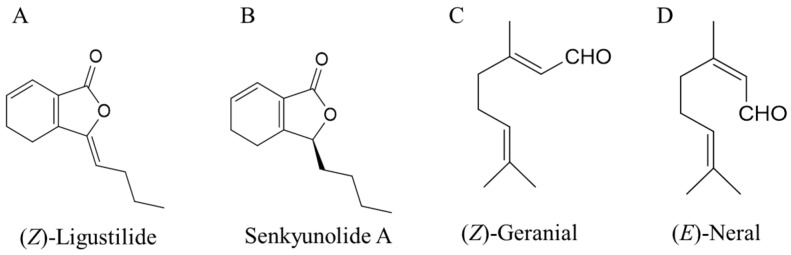
Structures of (*Z*)-ligustilide and senkyunolide A from *Ligusticum chuanxiong* oil, and (*Z*)-Geranial and (*E*)-Neral from lemongrass oil.

The list of principal compounds identified from LCO and LO is given in Table 2. By comparing the mass spectra data of the sample with literature data, eight main compounds of LCO were identified as (*Z*)*-*ligustilide (Figure 1A), senkyunolide A (Figure 1B), neocnidilide, 3-*n*-butylphthalide, butylidenephthalide, β-selinene, 1,3,5-undecatriene, and (*E*)-ligustilide. Meanwhile, eight main compounds of LO were identified as (*Z*)-geranial (Figure 1C), (*E*)-neral (Figure 1D), β-myrcene, geranyl acetate, terpinolene, isopulegol, trans-bergamotene, and citronellal.

### 2.3. Cooperative Virulence Index (c.f.) of LCO and LO

Bioassay of the mixture of LCO and LO was carried out, and the c.f. values of the mixtures were determined. The ratio of the mixture LCO–LO was 1:1 (*v*:*v*). The mixture was diluted with water X 1500 times, and the final concentration of LCO and LO were both about 66.7 mg/L. Data in Table 3 indicates that the mixture of LCO and LO has a synergistic effect, with a c.f. value of 23.17.

### 2.4. Confirmation of the Best Proportion of the Mixture

On the basis of the results above, a series of tests were carried out to confirm the optimal proportion of LCO and LO. Table 4 shows that when the LC_50_ ratios of LCO to LO were 80:20 and 90:10 (3.06:1 and 6.88:1 (*w*:*w*), respectively), a relatively high poison ratio of 1.22 and 1.21 was obtained. According to the characteristic of EC preparations, the optimum efficient and effective ratio of *L. chuanxiong* oil and lemongrass oil was established at 4:1 (*w*:*w*).

### 2.5. CTC Value of L. chuanxiong Oil and Lemongrass Oil

According to the results above, 20% LCO·LO EC (containing 16% LCO and 4% LO) was designed and prepared. Bioassays of 20% LCO EC, 20% LO EC, and 20% LCO·LO EC against *A. citricola* were conducted. Table 5 shows that, compared with LCO and LO, the control effect of 20% LCO·LO is more significant with a LC_50_ of 61.09 mg/L, which indicates that this formula has a synergistic effect, with a CTC value >180.

### 2.6. Preparation and Quality Test of 20% LCO·LO EC

Formulation of 20% LCO·LO EC was confirmed after the screening of solvents and emulsifiers. Then quality tests and bioassays were conducted according to the GB/T1603-79(89) standard. The formulation was a single-phase transparent liquid (pH 6.46), and there was no floating oil or sediment, which satisfied emulsification level II. After cold (0 °C) and thermal storage (54 °C) for 7 and 12 days, respectively, the preparation remained a single-phase transparent liquid. From Table 6, a relatively high activity of 20% LCO·LO EC against *A. citricola* after the thermal storage treatment was obtained. Moreover, a significant synergy with a CTC value >200 was still determined. This confirmed that thermal storage stability of 20% LCO·LO EC was excellent.

### 2.7. Field Trials of 20% LCO·LO EC against *Aphis citricola*

Field trial of 20% LCO·LO EC against *A. citricola* was carried out in Baishui county (Table 7) and Yangling city (Table 8), Shaanxi province. The EC formula of essential oil mixture were diluted X 500, 1000, and 1500 times, and final applied concentrations of LCO and LO were 200, 100, and 66.7 mg/L, respectively. Table 7 and Table 8 indicate that 20% LCO·LO EC exhibited significant control of *A. citricola*. The control effects of 500 times dilution were 90.06% (Table 7) and 87.24% (Table 8), which are comparable to 1000 times dilution of 20% imidacloprid EC. Meanwhile, the control at 1000 times dilution of 20% LCO·LO EC was still more than 80% seven days after the treatment.

## 3. Discussion

This study has confirmed that both LCO and LO have significant bioactivity against *Aphis citricola* with LC_50_ values of 128.8 mg/L and 169.6 mg/L, respectively. A mixture of these two essential oils shows a synergistic effect. Based on the field trials, the 20% LCO·CO EC preparation could be used as an alternative to imidacloprid. However, only one species of aphid was tested in this study, and a wider control spectrum needs to be confirmed in field trials.

It has been shown that *L. chuanxiong* oil and lemongrass oil are promising as pesticides and as activities against various kinds of pests [10]. Kwon found that butylidenephthalide, a main component of LCO, showed significant acaricidal activity against *Dermatophagoides farinae* with a 24-h LD_50_ value of 6.77 µg/cm^2^, which is comparable to that of benzyl benzoate (8.54 µg/cm^2^) [23]. Chu found that (*Z*)-ligustilide and 3-butylidenephthalide showed pronounced insecticidal activity against *Sitophilus zeamais* (LD_50_ = 10.23 and 15.81 µg/adult, respectively) [24]. Ricci et al. found that lemongrass oil could effectively repel the Russian wheat aphid, *Diuraphis noxia* (Mordvilko) [25]. Both of these oils could be developed as biopesticides [26]. However, there are few studies that have focused on the combined activities of these two oils against aphids.

Modes of action of these two essential oils and monomers have been investigated for several years. Modern phytochemical studies have shown that (*Z*)-ligustilide is the main lipophilic component of Danggui [27]. Yan has proved that (*Z*)-ligustilide is useful in the treatment of neurodegenerative disorders in which oxidative stress and apoptosis are mainly implicated [28]. Wang found that senkyunolide is an active component of *L. chuanxiong*, traditionally used to treat migraines, and the mechanism is through adjusting the levels of monoamine neurotransmitters and their turnover rates, as well as decreasing nitric oxide levels in the blood and brain [29]. (*Z*)-Geranial has antimicrobial activity against *Cronobacter* *sakazakii*, and it exerts its effect by inducing changes in ATP concentration, cell membrane hyperpolarization, and reduction in cytoplasmic pH [30]. Miron found that (*E*)-neral showed antifungal activities and an affinity for ergosterol, relating their mechanism of action to cell membrane destabilization [31]. From the pharmaceutical point of view, the synergy mechanism of essential oil was suggested as (1) a multi-target effect in which compounds target different sites; (2) pharmacokinetic or physicochemical effects on improved solubility or bioavailability; or (3) interactions of agents with resistance mechanisms [32]. In this study, we proposed that the synergistic effect of LCO and LO might be caused by a multi-target effect and improved solubility or bioavailability. In field trials, we found that the efficacy of the mixture is increased with time after application. This might be for the reason of slow-release properties of botanical pesticides.

Plant essential oil insecticides have been accepted for their high bioactivities and specific modes of action [10]. Recent investigations indicate that some chemical constituents of essential oils interfere with the octopaminergic nervous system of insects [33]. As this target site is not shared with mammals, these essential oil chemicals have low toxicity to non-target organisms and could be developed as biorational pesticides. This special regulatory status combined with a wide availability of essential oils in the flavor and fragrance industries has enabled the fast-track commercialization of essential oil-based pesticides in USA [34].

The need for chemical standardization and quality control is still a main barrier for the commercialization of essential oil-based pesticides [35,36]. Table 6 confirms the synergistic action that all parameters of LCO·LO EC and the heat stability of the active ingredient act against the target insect in the LCO·LO EC formulation.

Botanical pesticides, especially plant essential oils, would be advantageous in terms of pest resistance and behavior desensitization as they are the mixtures of natural compounds, acting synergistically against pest insects [35,37]. However, quality control is an urgent issue for botanical pesticide registration [26]. In this study, the stability of the bioactive principle in botanical pesticides is another important parameter for botanical pesticide registration.

In conclusion, our study showed that LCO and LO displayed significant bioactivities against *A. citricola*. Based on this, we have developed an essential oil-based pesticide that effectively controls *A. citricola* in the field. Thus, these two oils are worthy of development as novel biorational pesticides.

## 4. Materials and Methods

### 4.1. Materials

*Aphis citricola* were collected from apple trees at the Horticulture College of Northwest A & F University.

LCO and LO were purchased from Guangzhou Hengxin Flv. & Frag. Co., Ltd. (Guangzhou, China), and a 20% emulsifiable concentrate formulation (EC) was prepared for this investigation.

Imidacloprid EC with a concentration of 20% was purchased from Henan Planck Bio-Chemical Industry Co., Ltd., (Zhengzhou, China).

All the accessory ingredients, solvents, and other reagents were industrial products, which were purchased from Aladdin Industrial Corporation, Shanghai, China.

### 4.2. Bioassay

Essential oils were dissolved in acetone to a concentration of 100 mg/mL, and then diluted with 0.05% Tween 80 into stock solutions at a concentration of 10 mg/mL. Working solutions were diluted from stock solutions with water. Final concentrations of LCO and LO prepared for the bioassay were set at 25, 50, 100, 200, and 400 mg/L. Controls were 0.05% Tween 80 solutions with acetone at the same concentration as the treatment solutions. Clean apple leaves were collected and cut into leaf discs (each leaf disc was containing about 15~20 aphids). Each disc (3.5 cm in diameter) was dipped into the working solutions of essential oils for 10 s with each leaf disc receiving >100 μL of the solution. The discs were then air-dried in glass Petri dishes (9 cm in diameter) at room temperature. After the treatment, each leaf disc was placed individually in a covered glass Petri dish, and then incubated at a temperature of 25 °C with 14:10 h light:dark. Mortality was counted after 24 h with three replicates for each treatment.

### 4.3. Gas Chromatography-Mass Spectrometry (GC-MS) Analysis

The two essential oils—LCO and LO—were analyzed by a capillary GC-MS (Model GCMS-QP2010, Shimadzu, Kyoto, Japan). The gas chromatographic conditions were as follows: GC was oven fitted with a DB-5 MS 30 m capillary column (0.25 mm I.D., 0.25 µm film, thickness, Aglient, Palo Alto, CA, USA) with carrier gas helium at a flow rate of 1.2 mL/min, operating under an initial temperature at 80 °C for 2 min up to 250 °C in ramp rate of 10 °C/min, and then held for 11 min. A 1.0 µL sample solution was injected into the system with a split ratio of 1:50 for analysis. The electron impact ionization mass spectrometer was operated with an ionization voltage of 70 eV and an ion source temperature at 200 °C, using a scan mode and measuring the total ion chromatogram (TIC) under a mass range of 50.0–350.0. The datum analysis was performed on a NIST library [38,39] (Shimadzu, Kyoto, Japan).

### 4.4. Joint Action of the Two Essential Oils

Mixture of LCO and LO at their respective LC_50_ (128.78 and 169.59 mg/L) was prepared, and the bioactivity against *Aphis citricola* was determined using the above-mentioned bioassay method. The joint actions of the two essential oils were measured with the following equation according to Mansour’s method [40]:

(1) $$c.f.\;\mathrm{value}=\frac{\mathrm{observed}\;\%\;\mathrm{mortality}-\mathrm{expected}\;\%\;\mathrm{mortality}}{\mathrm{expected}\;\%\;\mathrm{mortality}}\times 100\;$$

When the cooperative virulence index (c.f.) of the mixture was >20, the mixture showed a synergistic effect. Similarly, when the c.f. was <−20, an antagonistic effect was shown. In the range of c.f. between −20 and 20, there was only an additive effect.

### 4.5. Determination of Efficiency Ratio and the Synergistic Effect

Following Zhang’s method [41], serial mixtures were prepared at a LC_50_ ratio (LCO–LO) of 10:0, 9:1, 8:2, 7:3, 6:4, 5:5, 4:6, 3:7, 2:8, 1:9, or 0:10. The poison ratios were calculated with following equations:

(2) $$\mathrm{Poison}\;\mathrm{ratio}\;\mathrm{of}\;a\;\mathrm{mixture}=\frac{\mathrm{Actural}\;\mathrm{mortality}}{\mathrm{Theorotical}\;\mathrm{mortality}}\times 100\;$$

with a poison ratio >1 defined as a synergistic effect, poison ratio <1 as an antagonistic effect. The highest poison ratio value of the two oils indicated the optimum synergistic ratio of LCO–LO. The virulence regression lines and median lethal concentrations (LC_50_) of the monomers and mixtures were obtained.

The co-toxicity coefficient (CTC) values of the mixtures were calculated by the method of Sun using the following equation [42], with CTC > 120 defined as a synergistic effect, CTC < 80 as an antagonistic effect, while any CTC values that fall into the range of 80–120 indicates additive effects. The ratio of 4:1 (LCO:LO, *w*:*w*) was selected for the 20% LCO·LO EC formulation.

(3) $$\text{CTC of a mixture}=\frac{\text{Actural Toxicity Index of a mixture}}{\text{Theoretical Toxicity Index of a mixture}}\times 100$$

### 4.6. Studies of EC Formulations of 20% LCO·LO

Optimum ratios of oil and surfactant were determined according to Wiwattanapatapee’s method [43]. The most suitable formulation that gave a clear homogeneous liquid with a stable emulsion after 1:20 dilution in water were obtained, and the ones with the best characteristics were selected for the preparation of the LCO·LO 20% EC.

The LCO·LO 20% EC was prepared by simple mixing [43]. The oil mixture (16 g of LCO and 4 g of LO) was mixed with a pesticide emulsifier 1601 (10 mL) using a mortar and pestle to obtain a homogeneous concentrate mixture, and the volume was then brought up to 100 mL with ethyl acetate. Thus, 20% LCO·LO EC (*w*/*v*) was prepared. The formulation was stored at room temperature (25 ± 3 °C) and protected from light.

Evaluation of the physical properties of 20% LCO·LO EC was processed according to Wiwattanapatapee’s method [43]. EC (2.5 g) was dispersed in 50 mL of distilled water via stirring with a magnetic stirrer at 500 rpm for 5 min at room temperature (25 ± 3 °C) for emulsification. The pH of the emulsion was also determined with a pH meter (Mettler-Toledo Co., Ltd., Guangzhou, China).

According to the GB/T1603-79(89) standard, the stabilities of the emulsion after cold storage (0 °C, 7 days) and thermal storage (54 °C, 12 days) were determined, respectively.

### 4.7. Field Trials of the Preparations

In October 2008, the field trials of the preparation against *A. citricola* were carried out in an apple orchard with a size of about 1 ha in Baishui, Shaanxi. Five-year host plants with heights of 1.2–1.5 m were selected. The field trials were conducted when the *A. citricola* infestation level was moderate. In June 2012, the field trials were carried out in an apple orchard with a size of about 1 ha in Yangling, Shaanxi. The treated plants were five years old, 1.2–1.6 m high, and highly infested.

Field trials were carried out according to GB/T 17980.9-2000, and trials were designed as follows: The prepared EC formulation was diluted 500, 1000, and 1500 times, and then applied via a constant spraying method (spray volume of 600 L/ha). In addition, 20% imidacloprid EC (1000 times dilution) was applied as a positive control. Each treatment was replicated three times. Water alone was applied as a negative control. Ten apple plants were set as one plot, and the plots were arrayed randomly. In each plot, 2 host plants were selected and marked, on which 5–10 leaves were marked and numbers of aphids on the leaves were recorded (over 200 aphids in every replicate). Numbers of live aphids on the marked leaves on the 1st, 3rd, and 7th days after spraying were counted. Then, the mortality of aphids and the control effect were calculated according to Abbott’s formula [44].

### 4.8. Statistical Analysis

The corrected control effect was transformed against the negative control and then processed for statistical analysis using the SPSS software (version 19.0, IBM, Armonk, NY, USA). Probit analysis was used to calculate LC_50_ (concentration causing 50% mortality compared with the control) values and their confidence intervals [45]. Statistical analysis in this study was processed with a SPSS 19.0 one-way ANOVA to assess the significance of difference between groups. The acceptance level of significance was *p* < 0.05. The results were expressed as mean ± S.D.

## Figures and Tables

**Table 1:** Toxicity of *Ligusticum chuanxiong* oil (LCO) and lemongrass oil (LO) against *Aphis citricola* van der Goot (24 h).

Pesticide	LC-P (*Y*=)	LC_50_ (mg/L)	r	Confidence Interval of LC_50_ (*p* < 0.05)	*χ*^2^
20% LCO EC	−5.55 + 4.99*x*	128.78	0.9822	116.44~142.42	5.45
20% LO EC	−6.56 + 5.18*x*	169.59	0.9922	162.75~169.71	3.71

LC_50_ value was determined by log-probit analysis.

**Table 2:** Identification and quantification of principal compounds from *Ligusticum Chuanxiong* oil and lemongrass oil in gas chromatography–mass spectrometry (GC-MS).

*Ligusticum chuanxiong* Oil		Lemongrass Oil	
Compound	Relative Content (%)	Compound	Relative Content (%)
(*Z*)-ligustilide	44.58	geranial	42.16
senkyunolide A	26.92	neral	32.58
neocnidilide	6.21	β-myrcene	4.95
3-*n*-butylphthalide	4.86	geranyl acetate	4.28
butylidenephthalide	2.95	terpinolene	1.58
β-selinene	2.15	isopulegol	1.38
1,3,5-undecatriene	1.83	*trans*-bergamotene	0.85
(*E*)-ligustilide	1.56	citronellal	0.38

**Table 3:** Cooperative virulence index (c.f.) of the mixture of *Ligusticum chuanxiong* oil (LCO) and lemongrass oil (LO) against *Aphis citricola* van der Goot (24 h).

Combinations	Concentration of Components	Theoretical Mortality (%)	Actual Mortality (%)	c.f.
LCO–LO (ratio 1:1, *v*:*v*)	66.7 mg/L	67.08	82.62 ± 3.18	23.1727

The blending ratio of LCO and LO was 1:1 (*v*:*v*).

**Table 4:** Toxicity of mixtures with different LC_50_ ratio of *Ligusticum chuanxiong* oil (LCO) and lemongrass oil (LO) against *Aphis citricola* van der Goot (24 h).

LCO LC_50_ (%)	100	90	80	70	60	50	40	30	20	10	0
LO LC_50_ (%)	0	10	20	30	40	50	60	70	80	90	100
Actual mortality (%)	52.11	62.5	62.62	56.31	51.94	54.76	49.46	51.09	48.02	48.5	48.02
Theoretical mortality (%)	52.11	51.70	51.29	50.88	50.47	50.07	49.66	49.25	48.84	48.43	48.02
Poison ratio	1	1.21	1.22	1.11	1.03	1.09	1.00	1.04	0.98	1.00	1

LC_50_ values of LCO and LO were 128.78 and 169.59 mg/L, respectively. In this study, the LC_50_ values of LCO and LO were set at 130 mg/L and 170 mg/L, respectively.

**Table 5:** Co-toxicity coefficient (CTC) value of 20% LCO·LO EC to *Aphis citricola* van der Goot.

Pesticide	LC-P (*Y*=)	LC_50_ (mg/L)	Confidence Interval of LC_50_ (*p* < 0.05)	r	*χ*^2^	CTC
20% LCO EC	−5.55 + 4.99*x*	128.78	116.44~142.42	0.9822	5.45	-
20% LO EC	−6.56 + 5.19*x*	169.59	162.75~169.71	0.9922	3.71	-
20% LCO·LO EC	7.42 + 1.99*x*	61.09	51.56~72.36	0.9768	2.03	221.46

LC_50_ value was determined by log-probit analysis.

**Table 6:** Toxicity of 20% LCO·LO EC before and after hot storage to *Aphis citricola* van der Goot (24 h).

Pesticide	LC-P (*Y*=)	LC_50_ (mg/L)	r	Confidence Interval of LC_50_ (*p* < 0.05)	*χ*^2^	CTC Value
20% LCO·LO EC After heating storage test	0.62 + 2.42*x*	64.32	0.9784	55.16~75.01	2.67	210.34

LC_50_ value was determined by log-probit analysis.

**Table 7:** The result of field trials of 20% LCO·LO EC on *Aphis citricola* van der Goot (October 2008, Baishui, Shaanxi).

Pesticide	Concentration of Components (mg/L)	Corrected Efficacy (%)		
		1 Day after Application	3 Days after Application	7 Days after Application
20%LCO·LO EC	200	46.08 ± 5.01 b	60.34 ± 4.32 b	90.06 ± 3.23 b
	100	43.99 ± 3.95 b	58.87 ± 3.08 b	80.76 ± 5.28 c
	66.7	37.45 ± 4.06 b	55.84 ± 5.36 b	63.88 ± 4.95 d
20% Imidacloprid EC	100	99.04 ± 5.36 a	100.0 ± 0 a	100.0 ± 0 a

Data are the mean of three replicates (50 aphids per replicate) and are represented as mean ± standard deviation. Means in the same column followed by the same lower case letter are not significantly different (*p* < 0.05) in a Tukey test.

**Table 8:** The result of field trials of 20% LCO·LO EC on *Aphis citricola* van der Goot (June 2012, Yangling, Shaanxi).

Pesticide	Concentration of Components (mg/L)	Corrected Efficacy (%)		
		1 Day after Application	3 Days after Application	7 Days after Application
20% LCO·LO EC	200	50.28 ± 5.63 b	63.31 ± 5.39 b	87.24 ± 1.62 b
	100	47.67 ± 4.16 b	60.56 ± 4.87 b	80.69 ± 2.31 c
	66.7	32.56 ± 4.01 c	49.23 ± 3.18 c	60.47 ± 4.28 d
20% Imidacloprid EC	100	97.76 ± 1.05 a	100.0 ± 0 a	100.0 ± 0 a

Data are the mean of three replicates (50 aphids per replicate) and are represented as mean ± standard deviation. Means in the same column followed by the same lower case letter are not significantly different (*p* < 0.05) in a Tukey test.
